# Neural Network–Based Algorithm for Adjusting Activity Targets to Sustain Exercise Engagement Among People Using Activity Trackers: Retrospective Observation and Algorithm Development Study

**DOI:** 10.2196/18142

**Published:** 2020-09-08

**Authors:** Ramin Mohammadi, Mursal Atif, Amanda Jayne Centi, Stephen Agboola, Kamal Jethwani, Joseph Kvedar, Sagar Kamarthi

**Affiliations:** 1 Northeastern University Boston, MA United States; 2 Partners connected for health Boston, MA United States; 3 Massachusetts General Hospital Department of Dermatology Boston, MA United States; 4 Harvard University Harvard Medical School Boston, MA United States

**Keywords:** activity tracker, exercise engagement, dynamic activity target, neural network, activity target prediction, machine learning

## Abstract

**Background:**

It is well established that lack of physical activity is detrimental to the overall health of an individual. Modern-day activity trackers enable individuals to monitor their daily activities to meet and maintain targets. This is expected to promote activity encouraging behavior, but the benefits of activity trackers attenuate over time due to waning adherence. One of the key approaches to improving adherence to goals is to motivate individuals to improve on their historic performance metrics.

**Objective:**

The aim of this work was to build a machine learning model to predict an achievable weekly activity target by considering (1) patterns in the user’s activity tracker data in the previous week and (2) behavior and environment characteristics. By setting realistic goals, ones that are neither too easy nor too difficult to achieve, activity tracker users can be encouraged to continue to meet these goals, and at the same time, to find utility in their activity tracker.

**Methods:**

We built a neural network model that prescribes a weekly activity target for an individual that can be realistically achieved. The inputs to the model were user-specific personal, social, and environmental factors, daily step count from the previous 7 days, and an entropy measure that characterized the pattern of daily step count. Data for training and evaluating the machine learning model were collected over a duration of 9 weeks.

**Results:**

Of 30 individuals who were enrolled, data from 20 participants were used. The model predicted target daily count with a mean absolute error of 1545 (95% CI 1383-1706) steps for an 8-week period.

**Conclusions:**

Artificial intelligence applied to physical activity data combined with behavioral data can be used to set personalized goals in accordance with the individual’s level of activity and thereby improve adherence to a fitness tracker; this could be used to increase engagement with activity trackers. A follow-up prospective study is ongoing to determine the performance of the engagement algorithm.

## Introduction

### Background

Studies have reported the efficacy of physical activity in reducing the risk of disease; however, physical inactivity is on rise in the United States [1]. Considering that physical inactivity was the fourth leading cause of mortality in 2016 [2], there is much emphasis on developing effective methods to maintain healthy levels of physical activity. One promising solution is wearable fitness trackers that enable individuals to monitor their activity levels and patterns to ensure a healthy level of physical activity [3].

It is reported that about 20% of the general health-tracking population use smart devices such as medical gadgets, mobile phone apps, or online tools to track their health data [4]. The use of technology to objectively monitor physical activity is associated with higher levels of activity [5]. However, the potential benefits derived from the use of physical activity trackers are challenged by the limited and transient adoption of devices that necessarily require sustained use to exert their intended effect. Continued engagement with fitness trackers is an issue that warrants further investigation [5] A previous study [6] found two factors associated with the adoption and sustained use of physical activity trackers: (1) the number of digital devices owned by the participants, and (2) the use of activity fitness trackers and other smart devices by the participants’ family members. The existence of these two factors bode well for the increased use of activity trackers; one study [1] demonstrated that motivational factors are associated with physical activity levels. Time constraints, fatigue, and aversion to exercise are some of the barriers to engaging in physical activity [1]. It has been reported that adjustments to activity targets are likely to enhance the users’ commitment to physical activity and engagement with fitness trackers [7].

With the rise of machine learning and availability of activity tracker data, it is possible to create a model that can learn from users’ behavior and adjust activity targets. Machine learning methods have broadly been applied to many health care areas such as cancer staging, risk assessment, and drug recommendation systems [8]. Researchers have studied the accuracy of activity trackers for energy expenditure assessment [9]. Having an automated personal trainer enabled by data mining techniques can be useful for an amateur athlete who cannot afford a personal trainer [10]. Similarly, effective feedback methods can be used for helping both athletes and coaches [11].

Although, there have been some attempts to study the benefit of activity trackers, there is room for studies on how to make use of activity trackers on a sustained basis. This study, to the best of our knowledge, is the first of its kind to develop a machine learning method to adjust the activity target for activity trackers. Machine learning techniques such as, but not limited to, lasso regression, ridge regression, Bayesian ridge regression, neural networks, random forest regression, and support vector regression have been used for prediction in the medical field [12].

Feature selection is an important step for improving model performance [13]. Prior to applying machine learning techniques, it is essential to study the data to find features that might negatively or positively affect the model [8]. In this work, we applied two feature selection techniques with a support vector machine [14]: principal component analysis [15] and recursive feature elimination.

**Figure 1:**
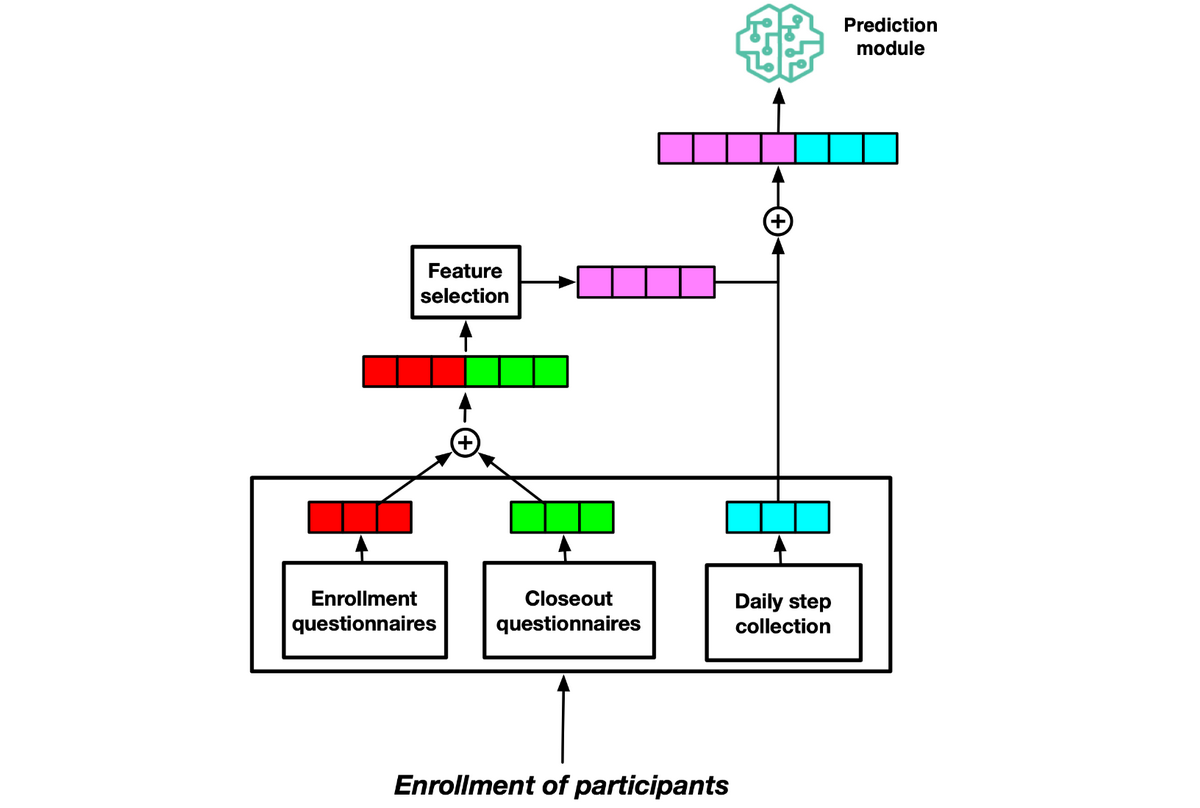
Study flow diagram.

We compared predictive models developed over (1) all features, (2) features generated by principal component analysis, (3) features selected by recursive feature elimination, and (4) features found from the authors’ previous study [6] that characterizes participants environments. Figure 1 shows the study flow diagram highlighting the key steps undertaken in this study.

### Objective

The purpose of this study was to develop a predictive model to estimate achievable weekly step goals. By setting realistic targets that are neither too easy nor too difficult to achieve, activity tracker users can be encouraged to continue to meet their weekly activity goals, and at the same time, to find utility of the tracker. We chose individuals who were overweight as our first use case, because the benefits of sustaining or even increasing physical activity in this population are well known, while there have been a lack of sustained interventions addressing this issue [16-18]. 

## Methods

### Data Collection

The study was designed as a 9-week, nonrandomized pilot in which the data were analyzed retrospectively. For this purpose, adults (N=30) with a BMI of 25 kg/m^2^ or greater were enrolled from a local Massachusetts General Hospital–affiliated clinic. After screening the participants and seeking their consent, the research team directed the study participants to visit the Wellocracy website [19] to read information regarding the study and review different types of activity trackers (and their features) available for their use during the 9-week study. The study staff assisted the participants with the device setup process as needed. For the study, 27 participants chose to use the FitBit Charge (Fitbit Inc) model, 2 chose the FitBit One (Fitbit Inc), and 1 chose the FitBit Zip (Fitbit Inc); 10 participants data were not used for analysis (7 participants either did not use the activity trackers at all or used less than 3 days a week, and an additional 3 participants demonstrated irregular use of their activity trackers on week by week basis). The data from the remaining 20 participants were used for model building. 

The following surveys were collected from each participant both at enrollment and at the closeout stages: Behavioral Regulation in Exercise Questionnaire (BREQ-2) [20]; Barriers to Being Active (BBA) [21]; Patient Health Questionnaire (PHQ-8) [22]; Prochaska Stage of Change [23]; and Patient-Reported Outcomes Measurement Information System (PROMIS) Global-10 [24]. These surveys included questions about technology use and the ownership of electronic devices (BREQ-2), questions related to perceived barriers to exercise and activity (BREQ-2 and BBA), depression screening questions (PHQ-8), stages of change (Prochaska), and general health questions (PROMIS Global-10).

The BREQ-2 is designed to gauge the extent to which reasons for exercise are internalized and self-determined based on the following categories: motivation, external, introjected, identified, and intrinsic. In contrast, the BBA assesses whether participants gauge certain categories as reasons for inactivity and includes energy, willpower, time, and resources. A score of 5 or greater for a category indicates that it is a substantial barrier to a person’s ability to exercise.

Participants were instructed to continuously wear the activity tracker for the entire period of the study. The first week was treated as a run-in period. Participants were contacted minimally during the remaining 8-week period to facilitate observation of participants’ activity tracker habits without interference. At the end of the study, participants completed a closeout survey either online or in paper format and underwent a phone interview to gather information on their experiences during the study. All interviews were conducted and transcribed for analysis by a trained neuropsychologist.

### Experimental Setting

We divided the data into disjoint training and testing sets. We chose 16 participants’ data as training data set and the remaining 4 participants’ data were used as the testing data set. We explored and fine-tuned hyperparameters using the training data set. Each participant was informed and had a fixed weekly activity goal for weeks 2 through 8; it was fixed for each participant at 110% of their week 1 average daily step count. Since we didn’t have a means to adjust the activity goal for each week for each participant, this value was used as an estimate of the personalized average daily step count goal for each week. We ignored the week if a participant used the tracker device for less than 3 days during the week.

### Data Preprocessing

We collected all data from questionnaires and the participants’ daily step count data from their activity trackers. The questionnaires generated 96 variables. The variables were screened to determine candidate predictors for building a machine learning model. In the first pass of the variable screening process, 11 variables whose variance was zero were eliminated. In the second pass, we examined pairwise correlations among all remaining variables to eliminate redundant ones. Since we found that all pairwise correlations were less than 0.6, we considered all variables to be nonredundant.

### Machine Learning Techniques

**Figure 2:**
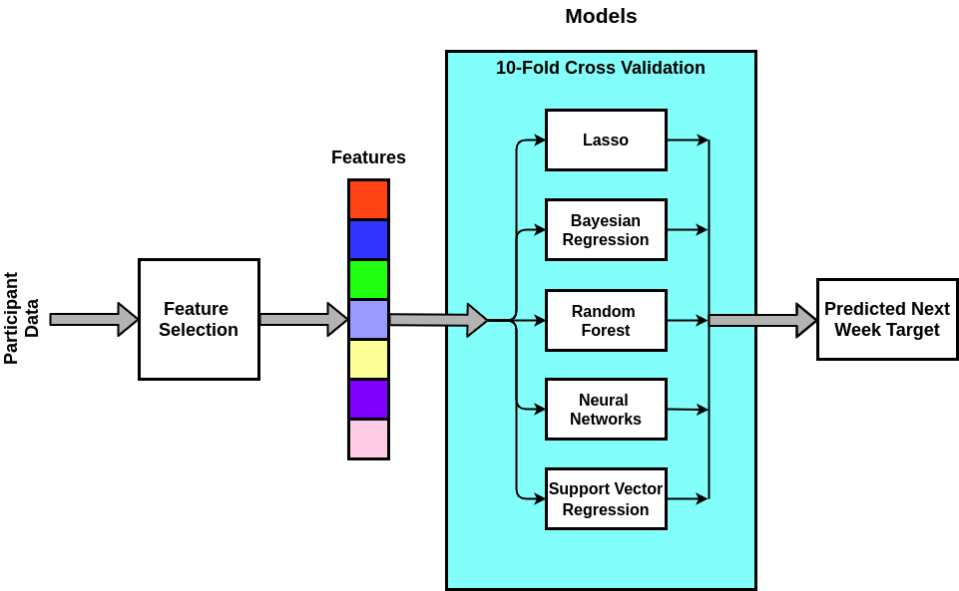
A schematic depicting models developed using the training data.

Figure 2 presents the models used to predict participants’ activity target. Selection of these models was based on their suitability and capabilities. Bayesian ridge regression estimates a probabilistic model of a ridge regression [25]. Lasso regression is robust to overfitting due to its regularization penalty [26]. Random forest models are versatile for numeric and categorical predictors and for classification and regression tasks. Random forest models can also be interpreted easily and are less susceptible to underfitting [27]. Neural network models consist of hundreds or thousands of neurons that perform mathematical operations to recognize patterns [28]. Support vector regression models are regression models whose optimization is unaffected by the dimensionality of the data [14].

We employed these models in four cases: (1) using all features, (2) using new features built by principal component analysis, (3) using important features found by recursive feature elimination, and (4) using a subset of features found from the authors’ previous study [6] that characterizes participants environments.

### Feature Selection and Extraction

In this study, we used participants’ mental health, behavioral information, and weekly activity performance as inputs. For every 7-day moving window, the subsequent 7 days were considered as the forthcoming week. The questionnaires provided a highly redundant and low variance data set. These features coupled with 7 daily steps counts of the week and a normalized Shannon entropy (*E_s_*) value of the weekly step count were considered candidate predictors for target step count for each individual participant for the forthcoming week [29]. The normalized Shannon entropy was computed as 
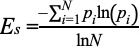
 where *i* denotes the day, *p_i_* denotes the portion of the total weekly steps completed on day *i*, and *N* denotes the number of days per week (ie, 7). The normalized Shannon entropy varies between 0 and 1. A value close to 0 indicates that the daily step counts of the participant throughout the week were irregular; in contrast, a value close 1 indicates that the step counts were consistent.

In total there were 93 candidate features for building the predictive models: 85 features from the questionnaires, 7 features from the daily steps of the week, and one weekly feature (ie, Shannon entropy). We used all 93 features for models developed in case 1.

For case 2, we developed a principal component analysis model. Principal component analysis is a dimension reduction technique that is widely used for extracting uncorrelated features components from correlated variables [14]. The principal component analysis used the 85 features from the questionnaires. We combined the principal components with the 7 daily and 1 weekly step count features.

In case 3, to identify the important features, we performed recursive feature elimination with support vector regression using the 85 questionnaire variables. We augmented these features with the 7 daily step count features and the normalized Shannon entropy value of the weekly step count.

Lastly, for case 4, we selected 2 features that were found to be important from the questionnaire in our previous work [6] studying the link between the participants’ environmental factors and the adherence to the use of activity trackers. These features were (1) the number of digital devices owned by the participants, and (2) the use of activity fitness trackers and other smart devices by the participants’ family members. We appended these two features with the daily step count features, and Shannon entropy of the weekly step count.

### Model Evaluation

We trained the models in this study with 10-fold cross-validation using the training data set. We compared the performance of the models using mean absolute error (MAE) and adjusted R^2^. We tested the final models (models A, B, C and D) of each case (cases 1, 2, 3, and 4) on an unseen test data set.

### Statistical Analysis

We used Python (version 3.5.0) and R (version 3.4.1) for model development and statistical tests. We performed a one-sided paired *t* test (*P*<.05 were deemed significant) for statistical comparison between the models. The null hypothesis was that the mean error of two models were equal, with the alternative hypothesis that the candidate model had a lower mean error than the mean error given by the comparison model.

## Results

### Participant Characteristics

We present the characteristics of the study participants in Table 1. Among them, 10 participants were eventually removed from the study since they stopped using the activity trackers.

**Figure 3:**
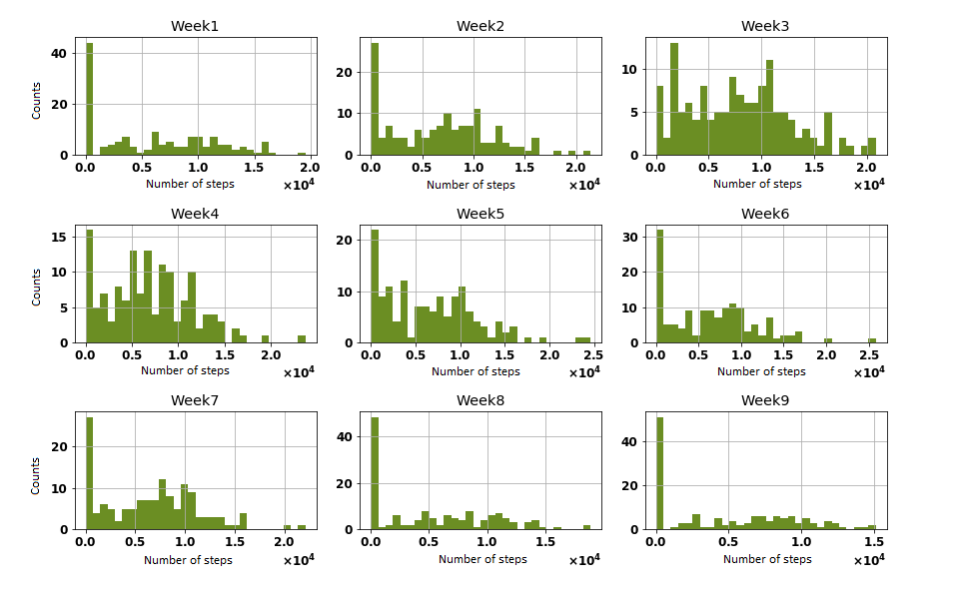
Weekly distribution of steps among all 20 participants.

**Figure 4:**
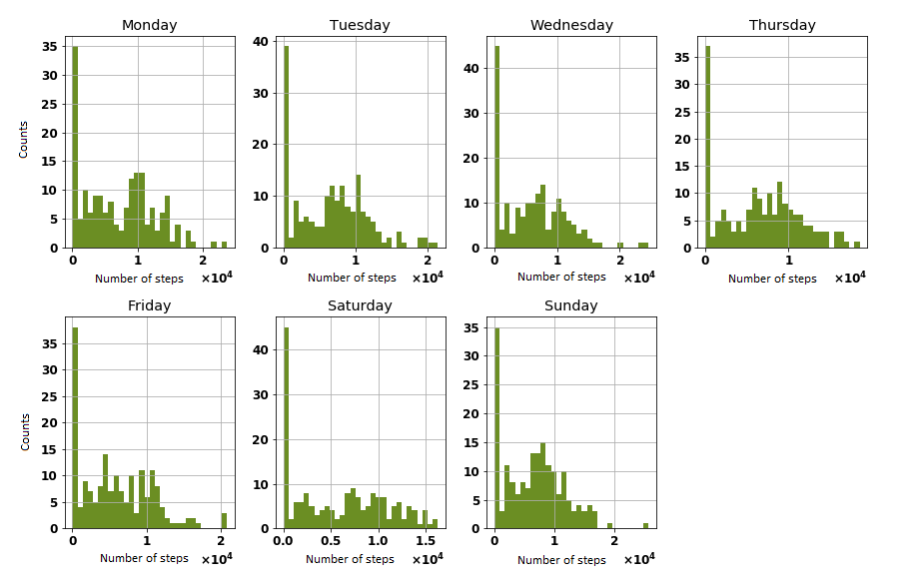
Step count distribution on each day of the week.

Over the span of 8 weeks, not all participants met their week-by-week step objectives. By and large, under half of participants met their progression objective every week (see Table 2). We present the distribution step count for all participants for each of the 9 weeks in Figure 3. We also presented the distribution of steps for each day of the week during the 9-week study period (see Figure 4).

**Table 1:** Patient demographic data.

Variable		Enrolled (N=30)		Participants (n=20)	
Age (years), mean (SD)		48.9 (9.5)		47.7 (10.2)	
**Gender, n (%)**					
	Male		9 (30)		6 (30)
	Female		21 (70)		14 (70)
**BMI at enrollment**					
	Mean (SD)		32.5 (4.6)		32.8 (4.7)
	Range		25.0-41.2		25.0-41.2
**Race, n (%)**					
	White		21 (70)		14 (70)
	American Indian or Alaskan Native		1 (3)		1 (5)
	Black or African American		3 (10)		2 (10)
	Hispanic		3 (10)		3 (15)
	Unknown		2 (6)		0 (0)
**Marital status, n (%)**					
	Married		8 (27)		6 (30)
	Divorced or separated		8 (27)		5 (25)
	Single (never married)		8 (27)		6 (30)
	Living with partner		3 (10)		2 (10)
	Widowed		1 (3)		1 (5)
	No response		2 (7)		0 (0)
**Education, n (%)**					
	12 years or completed high school or GED		5 (17)		3 (15)
	Some college		5 (17)		2 (10)
	College graduate		9 (30)		8 (40)
	Post–high school		2 (7)		2 (10)
	Postgraduate		2 (7)		1 (5)
	Less than high school		3 (10)		2 (10)
	Unknown		4 (13)		1 (5)
**Employment status, n (%)**					
	Employed/self-employed		15 (50)		12 (60)
	Disabled		5 (17)		3 (15)
	Unemployed		5 (17)		2 (10)
	Student		1 (3)		1 (5)
	Retired		1 (3)		1 (5)
	Unknown		3 (10)		1 (5)

**Table 2:** Participants meeting their average daily step goal for the week (110% of the average daily step count in week 1) over the course of the study.

Week	Participants (n=20) who met goal, n (%)
2	4 (20)
3	10 (50)
4	9 (45)
5	4 (20)
6	8 (40)
7	6 (30)
8	4 (20)
9	5 (25)

### Parameter Tuning and Feature Selection

**Figure 5:**
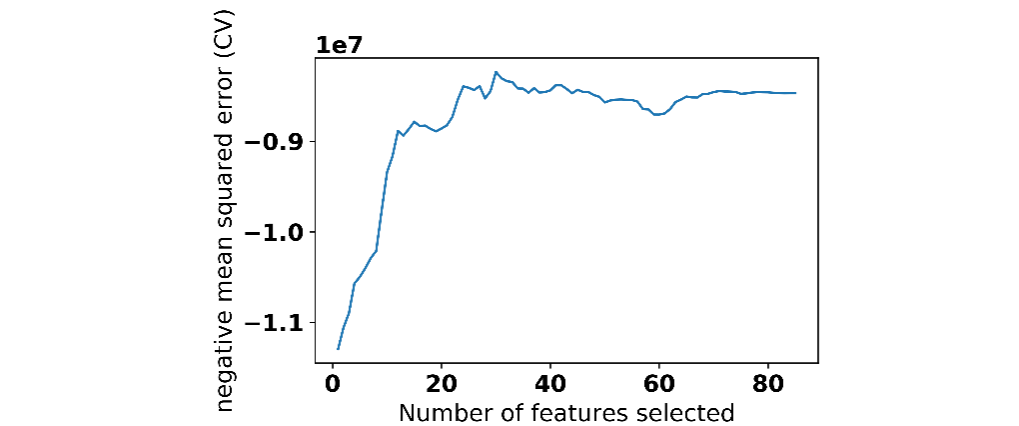
Number of features found to be important by support vector regression.

**Figure 6:**
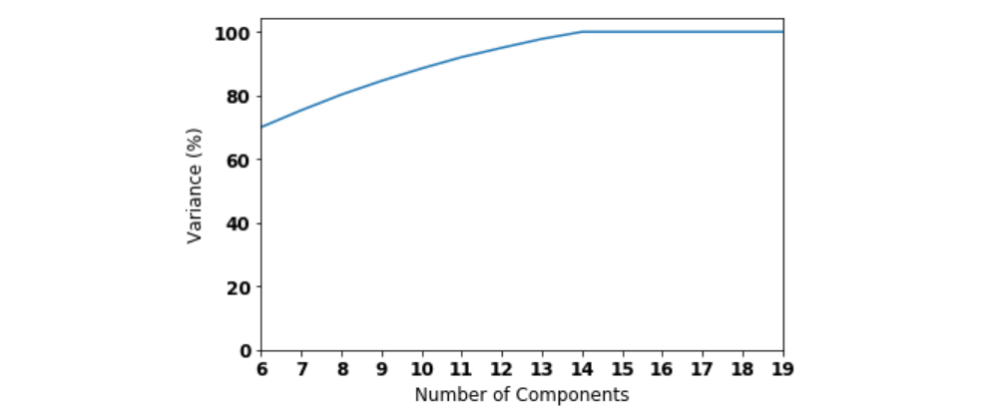
Number of components found by principal component analysis.

We performed a grid-search with 10-fold cross validation over the training data set. Lasso regression with α=0.01, Bayesian ridge regression with α=0.0001, and support vector regression with polynomial kernel of degree 2 with a regularization parameter of 0.00001 gave the best performance. Optimal parameters for random forests and neural networks depend on feature selection techniques. We found 30 variables from the questionnaires to be important using recursive feature elimination with support vector regression (see Figure 5). In the case of principal component analysis, the top 14 principal components explained 100% of the variance of the variables in questionnaires as shown in Figure 6.

### Model Performance

**Figure 7:**
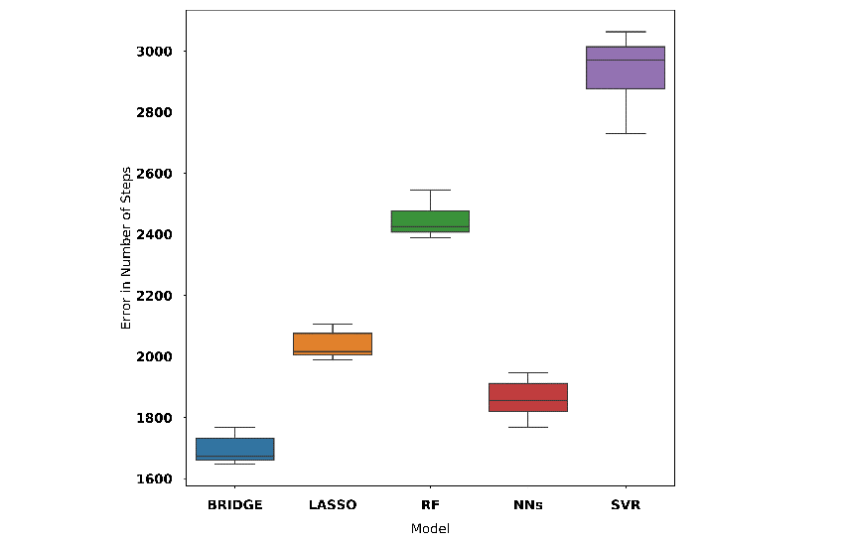
Cross-validation performance of models using all questionnaire features, 7 daily step count features, and weekly entropy feature. BRIDGE: Bayesian ridge regression; NN: neural network; RF: random forest; SVR: support vector regression.

We report the performance for all models of 10-fold cross-validation with the training data set in Table 3. We used mean absolute error (MAE) and adjusted R^2^ for the model comparison. Among all models developed, the Bayesian ridge regression model that used 93 features (case 1) gave the best performance for the training set (see Figure 7; MAE 1672, 95% CI 1640-1704; adjusted R^2^=0.85). The Bayesian ridge regression model is referred to as model A in the rest of this paper.

**Figure 8:**
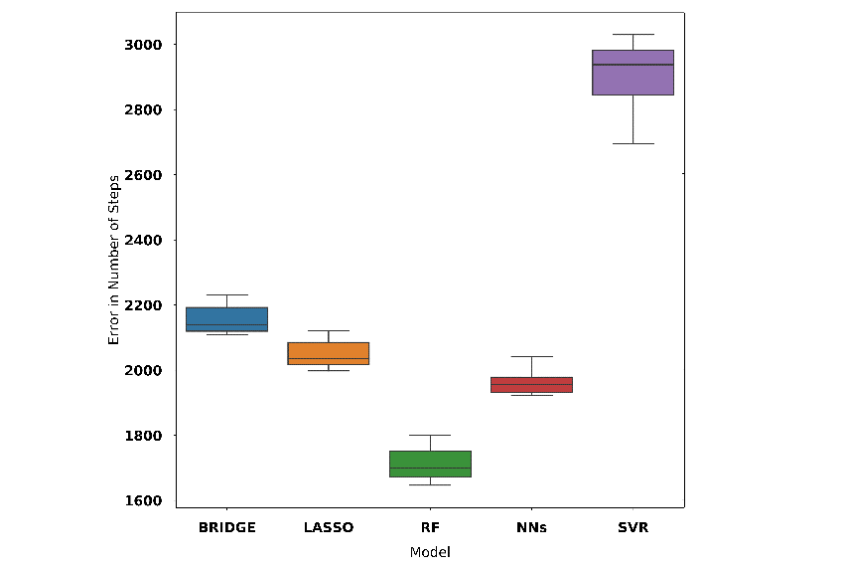
Cross-validation performance of models using features generated by principal component analysis, daily step count features, and weekly entropy feature. BRIDGE: Bayesian ridge regression; NN: neural network; RF: random forest; SVR: support vector regression.

Similarly, in case 2, we appended 14 principal components extracted from principal component analysis to the 7 daily and 1 weekly step count features resulting in 22 features. A random forest model (MAE 1700, 95% CI 1664-1737; adjusted R^2^=0.91) gave the best performance among all models (see Figure 8). This random forest model is referred to as model B for the rest of this paper.

**Table 3:** Results of 10-fold cross-validation with training set.

Case and models		Mean MAE		95% CI		*P* value	
				
**1 All features**							
	Bayesian ridge regression^a^		1672		1640-1704		Model ref^b^
	Lasso		2016		1985-2047		.002
	Random forest		2425		2386-2464		.002
	Neural network		1856		1813-1899		.002
	Support vector regression		2425		2386-2464		.002
**2 Feature selection using principal component analysis**							
	Bayesian ridge regression		2139		2107-2171		<.001
	Lasso		2036		2005-2067		<.001
	Random forest^c^		1700		1664-1737		Model ref
	Neural network		1956		1926-1985		.03
	Support vector regression		2938		2862-3013		<.001
**3 Feature selection using recursive feature elimination**							
	Bayesian ridge regression		2131		2100-2163		.002
	Lasso		2026		1995-2057		.002
	Random forest^d^		1774		1739-1809		Model ref
	Neural network		1906		1855-1958		.002
	Support vector regression		2548		2473-2624		.002
**4 Feature selected from previous study**							
	Bayesian ridge regression		2564		2537-2592		<.001
	Lasso		2457		2429-2485		<.001
	Random forest		1810		1768-1852		.04
	Neural network^e^		1622		1589-1655		Model ref
	Support vector regression		2940		2864-3016		<.001

^a^Model A (ie, this model gave the best performance in case 1).

^b^Reference model for comparisons.

^c^Model B (ie, this model gave the best performance in case 2).

^d^Model C (ie, this model gave the best performance in case 3).

^e^Model D (ie, this model gave the best performance in case 4).

**Figure 9:**
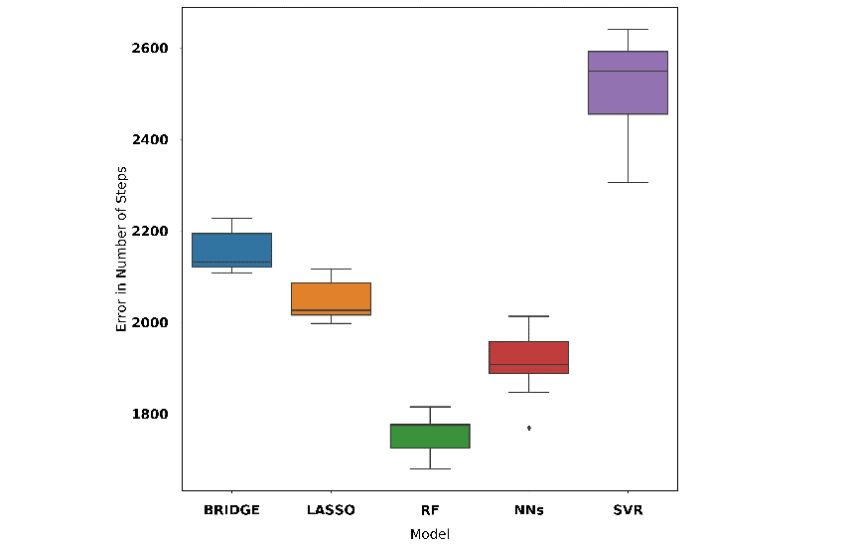
Cross-validation performance of models using all features given by recursive feature elimination, 7 daily step count features, and weekly entropy feature. BRIDGE: Bayesian ridge regression; NN: neural network; RF: random forest; SVR: support vector regression.

In case 3, we appended variables found by recursive feature elimination to the 7 daily step counts and 1 weekly entropy feature which resulted in 39 features. A random forest model (MAE 1774, 95% CI 1739-1809; adjusted R^2^=0.81) offered the best performance among all the models developed with these features (see Figure 9). We refer to this random forest model as model C.

**Figure 10:**
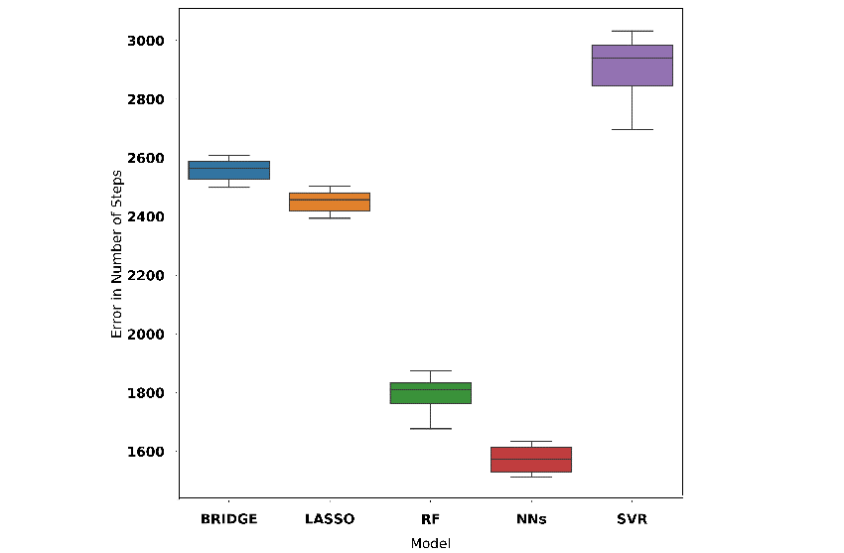
Cross-validation performance of models using features generated from previous knowledge, 7 daily step count features, and weekly entropy feature. BRIDGE: Bayesian ridge regression; NN: neural network; RF: random forest; SVR: support vector regression.

Finally, for case 4, we coupled two features found in the previous study [6] with the 7 daily step counts and 1 weekly entropy feature which resulted in 10 features. A neural network gave the best performance across all models (MAE 1622 steps, 95% CI 1589-1655; adjusted R^2^=0.89) (see Figure 10). We refer to this neural network model as model D.

**Figure 11:**
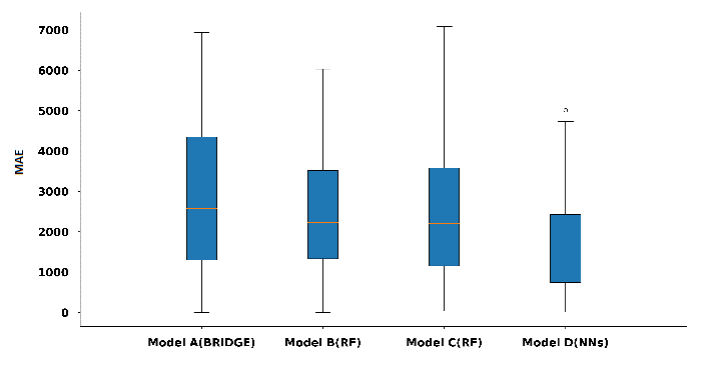
Boxplot of errors in terms of steps over the test set for Model A (Bayesian ridge regression), Model B (random forest), Model C (random forest), and Model D (neural network). MAE: mean absolute error.

Model D gave the best predictive performance among all the models. It had the lowest MAE across all models explored. We compared the predictive power of the model D (neural networks) with those of models A, B, and C using the testing data set. We found that model D gave a better predictive performance as shown in Figure 11. We performed comparisons using *t* tests between the errors generated by the model D and errors generated by models A, B, and C as shown in Table 4. We observed that model D’s lower errors in comparison to those of Bayesian ridge regression (model A: *P*=.01), random forest (model B: *P*<.001), and random forest (model C: *P*=.01) models were significant.

**Table 4:** Breakdown of model results over the test data set.

Model (type)	Mean MAE^a^	95% CI	*P* value
Model D (neural network)	1545	1383-1706	Model ref^b^
Model C (random forest)	2210	1990-2420	.01
Model B (random forest)	2230	2015-2445	<.001
Model A (Bayesian ridge regression)	2578	2310-2845	.01

^a^MAE: mean absolute error.

^b^Reference model for comparisons.

We evaluated the naïve rule: using participants’ average daily step count of the week as the prediction for the subsequent week’s activity goal [30]. This is a reasonably competitive approach because the weekly target exhibited strong autocorrelation. The naïve rule achieved an MAE of 1664 steps while model D achieved an MAE of 1545 steps (95% CI 1383-1706) for the 4 test participants over a period of 8 weeks.

### Sensitivity Analysis

Its reported that the Fitbit activity devices have margin of ±5% error in their step count readings [31,32]. We tested the performance of our best model (model D) on three noisy data sets generated from the original test data set by adding ±1%, ±3%, and ±5% noise. The first noisy data set was generated by adding random noise between –1% and +1% to the Fitbit readings. Similarly, the second and third noisy data sets were generated by adding ±3% and ±5% noise to the original data set. To conduct the sensitivity analysis, we generated 100 replications of each noisy data sets by adding different random noises with limit specified for each data set. These data sets were used to evaluate the performance of model D.

The model D achieved an MAE of 1606 steps (95% CI 1490-1755) for the noisy test data set generated using margin of ±1% error which is approximately 4% higher than the MAE achieved for the original noise-free test data set. Similarly, model D’s MAE was 1670 steps (95% CI 1571-1840) for the margin of ±3% error which was approximately 8% higher than the MAE achieved for the original noise-free test data set. Finally, for the data set generated using margin of ±5% error, model D’s MAE of 1710 steps (95% CI 1621-1908) was approximately 10% higher than the MAE achieved for the original noise-free test data set.

This led to the empirical observation that 2%, 6%, and 10% noise in Fitbit readings leads to approximately 4%, 8%, and 10% increases in prediction error, respectively. Therefore, we assume that the model error sublinearly increases with margin of error in activity tracker readings. However, one might need to validate this observation with further evaluation data.

### Feature Importance

**Figure 12:**
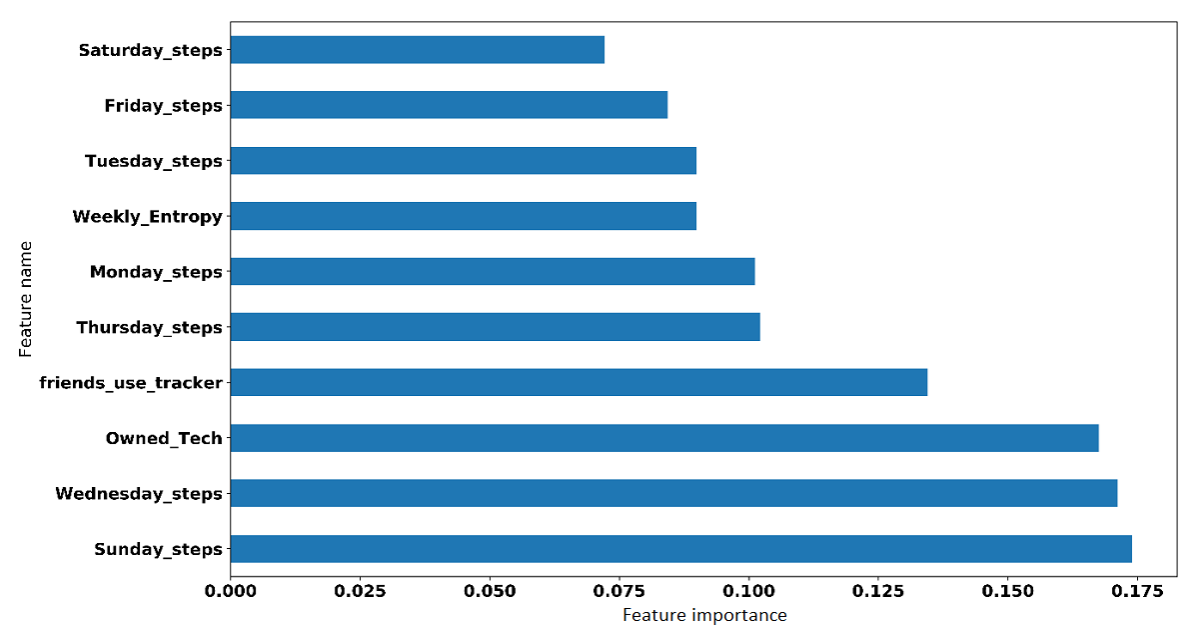
Importance of the features as measured by the integrated gradient method.

We experimented with integrated gradients [33] in order to analyze the features of model D. This method provides a score that reflects the contribution of each variable to the response variable by calculating the integral of the gradient of the response variable with respect to that variable. We report the top features for model D in Figure 12.

## Discussion

Precise prescient calculations that consolidate information are one of the main focuses in predictive analyses [34]. To the best of our knowledge, this work is one of the first studies to explore machine learning models with the aim of adjusting step count goals. Inputs to these models use an individual’s personal, social, and environmental factors as well as weekly activity data. A recent study concluded that activity tracker users feel unmotivated despite having knowledge about the benefits of physical activity [35]. Users can become unmotivated if they cannot meet their activity goal [36]. Step count goals that are too easy to achieve may lead to abandonment of the activity tracker, and those that are too high will discourage the individual [37].

In previous work [6], we studied the factors influencing the use of activity trackers and identified two factors that likely promote the continued use of activity trackers: (1) the number of digital devices that the participant owns, and (2) whether or not members of the participants’ family use activity fitness trackers and other smart devices. Extending the previous work [6], in this study, we explored different predictive models to estimate achievable weekly step goals to encourage the use of activity trackers. This study can be used to set goals for individuals and can be accompanied by proper motivation messages to improve the sustained use of the activity trackers [38-40].

This study has some limitations. The number of participants was low, and all of the participants were selected from a cohort with BMI of 25 kg/m^2^ or greater from the same geographical area. Participants who choose to participate in this study were more likely to use from 3 models of Fitbit activity trackers than those who chose not to participate in the study. Moreover, only participants who completed the 9-week study were considered for further analysis. Finally, in this study, we used 110% of week 1 averaged daily step count as the best estimate of each participant’s personalized goal for each week. The goal may have been on heavy side for some participants and on the easy side for others. This is, of course, a limitation of the study in the absence of a mechanism for a correct estimate of the goal for each participant.

As an extension to this work, a new study with the goal of validating the developed model with a large number of participants has been undertaken. The new study recruited 120 individuals from the general public to use the neural network-based predictive model developed herein over a period of 6 months. This model, hosted on a server, provides each user with achievable daily steps goals and updates the model parameters on a weekly basis. The data from the study that is underway will be used to fine tune the predictive model and to gain insight into activity tracker users to motivate and manage their physical activity.

## Abbreviations

BBA: Barriers to Being Active
BREQ: Behavioral Regulation in Exercise Questionnaire
MAE: mean absolute error
PHQ: Patient Health Questionnaire
PROMIS: Patient-Reported Outcomes Measurement Information System
